# Intraoperative Fungal and Acid-Fast Bacilli Culture Use in Orthopedic Surgery and the Cost to the Medical System: A Narrative Review

**DOI:** 10.7759/cureus.105961

**Published:** 2026-03-27

**Authors:** Rohan V Rajan, Joshua Gruber, Trevor Betros, Christopher Ferguson, Timothy Ackerman

**Affiliations:** 1 Osteopathic Medicine, Nova Southeastern University Dr. Kiran C. Patel College of Osteopathic Medicine, Davie, USA; 2 Orthopedic Surgery, UPMC Central PA, Harrisburg, USA; 3 Orthopedic Surgery, Allegheny Health Network, Pittsburgh, USA

**Keywords:** acid-fast bacilli, fungal cultures, healthcare costs, intraoperative cultures, orthopedic surgery, prosthetic joint infection

## Abstract

Fungal and acid-fast bacilli (AFB) infections are uncommon but clinically important causes of surgical site infection (SSI) and periprosthetic joint infection in orthopaedic surgery. Despite their rarity, fungal and AFB cultures are frequently obtained intraoperatively, and practice patterns vary widely due to the absence of broadly accepted, standardized guidance. This narrative review descriptively summarizes the available literature on fungal and AFB culture use in orthopaedic surgery, with a focus on reported indications for intraoperative testing, diagnostic yield, and cost considerations, in order to highlight practice variation and identify areas for future study. Across studies, standardized protocols for when and how to obtain fungal and AFB cultures were uncommon, and reported culture positivity was generally low, with many positive results representing contaminants or findings of uncertain clinical significance. Routine ordering in low-risk scenarios was associated with substantial cumulative cost, including annual institutional expenditures and multi-year charges in some cohorts. Targeted, protocol-driven approaches demonstrated meaningful reductions in low-yield testing, including reports of large decreases in unnecessary cultures in appropriately sized practice settings. Routine intraoperative fungal and AFB cultures in orthopaedic surgery appear to have low diagnostic yield in many commonly tested scenarios and often do not change clinical management, raising concerns about cost-effectiveness. A selective, indication-driven strategy, such as prioritizing higher-risk hosts, specific anatomic sites, and cases with clinical suspicion for atypical infection, may preserve diagnostic value while reducing unnecessary costs. Future work should focus on developing and validating standardized testing algorithms and evaluating long-term cost-benefit outcomes of targeted versus routine culture practices.

## Introduction and background

Infections remain one of the most common complications in surgical procedures. Specifically, surgical site infections (SSIs) and prosthetic joint infections (PJIs) are particularly consequential, contributing to increased morbidity, extended hospital stays, and substantial healthcare costs [1]. Perioperative infection prevention and management have traditionally focused on bacterial infections, such as *Staphylococcus aureus*, notably methicillin-resistant *S. aureus *(MRSA) being the most predominant [2]. Fungal and acid-fast bacilli (AFB) infections are far less common, but they carry a disproportionate clinical burden because of their diagnostic complexity, delayed recognition, and treatment challenges. In the arthroplasty literature, fungal PJIs have been reported to account for approximately 1%-3% of all joint infections, whereas mycobacterial PJIs are even rarer, with available series reporting rates of approximately 0.5% in hip and knee PJIs [3-6]. Despite their rarity, intraoperative fungal and AFB cultures are obtained in orthopedic surgery, often out of concern for atypical pathogens in indolent, recurrent, or otherwise complex infections and in the absence of widely accepted guidance regarding when such testing is truly warranted. At the same time, there is no widely accepted standardized approach for when these cultures should be ordered, and the cost implications of routine or low-yield testing remain poorly characterized. However, much of the existing discussion centers on arthroplasty, and intraoperative fungal and AFB cultures are also obtained across a broad range of orthopedic subspecialties, including trauma, spine, foot and ankle, and upper extremity surgery.

Although bacterial organisms account for the vast majority of PJIs [7], fungal and mycobacterial infections remain important diagnostic considerations in selected patients because they often present indolently and may be missed without atypical culture testing. As surgical volume continues to grow, even rare infections may impose increasing absolute burden, underscoring the need for more selective and evidence-informed diagnostic strategies [8]. However, the rarity of clinically meaningful fungal and AFB infection raises important questions about the diagnostic value and economic justification of routinely obtaining these cultures in orthopedic practice.

The objective of this narrative review is to descriptively summarize current literature on the use of intraoperative AFB cultures in orthopedic surgery, with emphasis on reported indications for testing, diagnostic yield, practice variation, and published cost considerations. This review is intended to identify recurring themes, highlight gaps in standardization, and generate hypotheses for future protocol development rather than serve as a comprehensive systematic evidence synthesis.

## Review

Methods

This study was conducted as a narrative review of the literature. Relevant publications were identified through searches of PubMed, Web of Science, EMBASE, and Ovid MEDLINE. The search was performed from database inception through January 2024 and used combinations of the following terms: “orthopedic surgery”, “orthopaedic surgery”, “fungal culture”, “acid-fast bacilli”, “AFB”, “mycobacterial culture”, “atypical culture”, “periprosthetic joint infection”, “surgical site infection”, “arthroplasty”, “revision arthroplasty”, “spine surgery”, “foot and ankle surgery”, and “cost”. Additional articles were identified through manual review of reference lists from relevant studies.

Studies were considered for inclusion if they reported on intraoperative fungal and/or AFB/mycobacterial cultures in orthopaedic surgical settings, including literature addressing clinical indications for obtaining cultures, microbiologic yield or positivity rates, interpretation of positive results, utilization patterns, protocolized testing strategies, or financial and resource-use implications. Case reports, retrospective cohort studies, cost analyses, and other clinically relevant reports were considered when they contributed to the practical understanding of fungal or AFB culture use in orthopaedic surgery. Studies not focused on orthopaedic surgery, not addressing intraoperative fungal or AFB culture use, or lacking relevance to the clinical questions of interest were excluded.

After retrieval, titles and abstracts were screened for relevance, and full texts of selected articles were reviewed in detail. Because this was a narrative review, studies were not selected using a formal Preferred Reporting Items for Systematic Reviews and Meta-Analyses (PRISMA) workflow, and no formal risk-of-bias assessment or quantitative meta-analysis was performed. Instead, studies were prioritized based on relevance to the review objectives, with emphasis on literature describing indications for culture acquisition, reported positivity and true infection rates, and economic or systems-level implications. Data elements abstracted from the reviewed literature included study setting, patient population, surgical context, culture acquisition details when available, microbiologic findings, and cost-related outcomes. Findings were synthesized qualitatively and organized by clinical scenario to highlight practical implications for fungal and AFB culture utilization in orthopaedic surgery.

Review

Indications for Obtaining Cultures

The decision to obtain intraoperative fungal and AFB cultures in orthopedic surgery is typically driven by clinical suspicion and perceived patient or procedure-specific risk, yet the literature offers limited consensus on which factors meaningfully increase the likelihood of clinically relevant results. Across published cohorts, immunosuppression and select anatomic sites appear more consistently associated with positive fungal cultures than routine arthroplasty or revision settings. Key study characteristics and reported outcomes are summarized in Table 1.

**Table 1:** Summary of studies on utilization and diagnostic yield of intraoperative fungal and/or AFB/mycobacterial cultures in orthopedic surgery Abbreviations: AFB = acid-fast bacilli; PJI = prosthetic joint infection; I&D = irrigation and debridement

Study	Setting/Cohort	Utilization (how often ordered)	N (patients/procedures/cultures)	Fungal positivity	AFB positivity	Key notes
Lambrechts et al. [9]	Spine irrigation & debridement (I&D)	Not reported	202 Patients	4%	0.49%	Steroid use and repeated I&D associated with a higher likelihood of positive fungal/AFB cultures
Sanderford et al. [10]	Orthopedic procedures (anatomic/risk-stratified)	Not reported	813 Patients eligible	3.7%	0.1%	Peripheral vascular disease associated with positive fungal cultures (OR 3.5); hand/foot surgery (OR 4.2)
Oetojo et al. [11]	Hip & knee arthroplasty (primary/conversion/revision)	Not reported	481 Patients, 858 procedures, 238 cultures	Combined fungal/AFB positivity: 5.8% overall; conversion 14.3%; aseptic revision 3.6%	Included in the combined rate	Higher positivity in immunosuppressed subgroups (HIV/AIDS: 33%, metastatic solid tumors: 20%, malignancy: 8.3%)
Golden et al. [12]	Suspected PJI	93.4% of patients had mycobacterial cultures ordered	97 Patients, 91 cultures	Not applicable	1.1%	No positives in the revision arthroplasties subset described
Contreras et al. [13]	Revision shoulder arthroplasty	Cultures sent in 158/237 procedures	237 Procedures; 187 fungal; 174 AFB cultures	0%	0%	AFB/fungal accounted for 53.2% of total culture charges (see Table 2)
Wadey et al. [14]	Revision arthroplasty/PJI evaluation	Protocol-driven (targeted rather than routine)	253 Procedures; 446 AFB + 486 fungal cultures (1 year)	0.6%	0%	Protocol: 1 representative tissue for full workup + 4 additional samples for aerobic/anaerobic only; 80% reduction in unnecessary cultures; projected ~$193,110/year savings (300 cases/year)
Tokarski et al. [15]	Presumed aseptic hip/knee revision arthroplasty	Routinely obtained	1717 Procedures; 2272 patients; 3111 fungal; 3106 AFB cultures	1.7%	0.5%	Supports low yield in aseptic revision arthroplasty
Dean et al. [16]	Foot & ankle surgery	High frequency (qualitative)	322 Patients; 525 fungal; 434 AFB cultures	4.2%	0%	Highlights that positivity does not necessarily reflect a clinically meaningful infection
Lee et al. [17]	Suspected PJI (sonicate vs tissue)	Not reported	713 Fungal cultures; 331 mycobacterial cultures	2.95%	3.02%	The authors concluded that routine fungal/mycobacterial cultures may not be necessary
Tai et al. [18]	Suspected PJI	Not reported	3,629 Fungal cultures; 2,923 mycobacterial cultures	5%	1.2%	Supports low yield of mycobacterial cultures; fungal positivity is higher, but still uncommon
Kazmers et al. [19]	Acute deep upper-extremity infections with purulence (surgical debridement)	Not reported	100 Patients; 100 procedures; 94 fungal; 82 AFB	5.3%	2%	Suggests yield may be higher in select acute deep infections

Host-related factors reported to increase the likelihood of positivity include immunosuppression and vascular comorbidity. In spine irrigation and debridement procedures, Lambrechts et al. found that steroid use was associated with a higher likelihood of positive fungal or AFB cultures in spine debridement procedures [9]. Similarly, Sanderford et al. identified peripheral vascular disease as a risk factor for positive fungal cultures, with an adjusted odds ratio (OR) of 3.5 [10].

Several comorbidities, including diabetes mellitus and immunocompromised states, are well-established risk factors for orthopedic infections broadly and have also been implicated in fungal PJI in prior literature [20,21]. However, it is important to distinguish general infection risk from risk specifically demonstrated within the studies included in this review. Among the reviewed studies, direct evidence supporting increased yield of intraoperative fungal or AFB cultures was limited and not uniform across all comorbid conditions. Rather, the most consistent associations within this review were seen with steroid-related immunosuppression, select immunocompromised subgroups, and peripheral vascular disease [9-11].

Immunocompromised patients, including those with HIV infection, are recognized as susceptible to PJIs and SSIs. Oetojo et al. supported this notion by finding that AFB and fungal cultures taken from patients with HIV or AIDS yielded a 33% positivity rate; other notable high positivity rates within the realm of immunosuppression included metastatic solid tumors (20%) and malignancy (8.3%) [11]. However, one significant limitation of the study is that these estimates were limited by small subgroup sample sizes and should be interpreted cautiously [11].

Revision arthroplasty is also generally considered a risk factor for infection. A large retrospective study of 476 knees found that the infection rate after revision total knee arthroplasty (TKA) was 9%, with a significantly higher rate in those revised for infection (21%) compared to those revised for aseptic failure (5%) [22]. However, no studies within our review identified revision procedures as a significant risk factor for infection by fungal or AFB species. Golden et al. documented that, out of 29 revision arthroplasties within the population of the study, there were no positive mycobacterial cultures [12]. Furthermore, Contreras et al. documented a total of 361 fungal and AFB cultures obtained in 237 arthroplasty procedures, yet none of the cultures were positive [13]. Collectively, these findings suggest that revision status alone may not be a sufficient indication for routine fungal/AFB cultures in the absence of additional clinical concern.

Finally, anatomic location may warrant a lower threshold for fungal cultures. Sanderford et al. reported that surgeries involving the hand or foot had higher odds of positive fungal cultures compared with other regions (adjusted OR: 4.2) [10], supporting a more selective, location-sensitive approach to ordering. Taken together, the current literature supports targeted fungal/AFB culture acquisition, prioritizing high-risk hosts, select anatomic sites, and scenarios in which atypical infection is clinically suspected, rather than routine testing across all orthopedic procedures.

Standardized Protocols

Despite the frequent ordering of fungal and AFB cultures in orthopedic practice, there is no universally accepted protocol guiding when and how these tests should be obtained. This lack of standardization contributes to variability in practice patterns and may increase low-yield testing, particularly in settings where positivity and “true infection” rates are consistently low. Wadey et al. described a protocol developed by surgeons that demonstrated the ability to significantly reduce healthcare funds spent on testing fungal and AFB cultures without affecting the ability to detect true pathogens on tissue samples; in this study, this protocol recommended that surgeons select one representative tissue sample for full bacterial, AFB, and fungal culture workup, while four additional samples are tested only for aerobic and anaerobic bacteria [14]. This approach not only streamlines the diagnostic process but also emphasizes the importance of targeted sampling in identifying the presence of less common organisms, such as mycobacteria, which can complicate postoperative recovery and lead to significant morbidity if not recognized. This is currently the only study suggesting a standardized diagnostic protocol for identifying these infections. Future work should evaluate whether protocolized algorithms can be tailored to specific procedures, such as arthroplasties, debridements, PJIs, and osteomyelitis, and whether targeted testing strategies maintain clinical outcomes while reducing laboratory burden and cost.

Frequency of Obtaining Cultures

Multiple studies demonstrate that fungal and AFB cultures are frequently obtained in orthopedic surgery, often as part of routine intraoperative practice rather than in response to a defined set of atypical infection criteria. In presumed aseptic hip and knee revision arthroplasty, Tokarski et al. reported that atypical cultures were routinely obtained [15]. Similarly, Oetojo et al. evaluated AFB and fungal cultures across a range of hip and knee arthroplasty procedures, reflecting broad use in arthroplasty settings [11]. High ordering frequency has also been observed in suspected PJI: Golden et al. reported that mycobacterial cultures were ordered for 93.4% of patients evaluated for suspected periprosthetic joint infection [12]. Dean et al. described similarly frequent ordering in foot and ankle surgery cohorts [16]. In revision shoulder arthroplasty, Contreras et al. highlighted the magnitude of utilization and its economic footprint, noting that fungal and acid-fast cultures accounted for 53.2% of total culture charges; among 237 procedures, cultures were sent for 158 surgeries, yielding 187 fungal and 174 acid-fast cultures [13]. These studies collectively indicate a widespread practice of obtaining fungal and AFB cultures in orthopedic procedures, although the clinical justification for this routine practice is under increasing scrutiny.

Incidence and Positivity Rates

Across orthopedic settings, reported fungal and AFB culture outcomes should be interpreted with careful distinction between raw culture positivity, true infection, and clinically meaningful infection. In this review, “culture positivity” is defined as any fungal or AFB growth reported on intraoperative specimens, whereas “true infection” refers to positive cultures judged by the original study authors to represent genuine infection rather than contamination or findings of uncertain significance. “Clinically meaningful infection” further refers to infections likely to influence diagnosis, treatment, or surgical decision-making. These outcomes are not interchangeable, and failure to distinguish among them may either overstate or understate the practical diagnostic value of atypical cultures.

Reported positivity rates for fungal and AFB cultures vary by anatomic site and clinical scenario; however, many cohorts demonstrate low yield, and “true infection” rates may be substantially lower than raw culture positivity due to contaminants or results without clear clinical significance. In a comprehensive arthroplasty study, Oetojo et al. reported a combined fungal/AFB culture positivity rate of 5.8%, with higher positivity in conversion procedures (14.3%) than in aseptic revisions (3.6%) [11]. In contrast, Tokarski et al reported extremely low rates in presumed aseptic hip and knee revisions, with a true-positive rate of 0% for AFB cultures and 0.1% for fungal cultures [15]. These findings illustrate that positivity rates may increase in more complex clinical contexts but remain very low in routine aseptic revision practice.

In suspected PJI cohorts, several studies demonstrate low positivity for fungal and mycobacterial cultures despite high testing volume. Lee et al. reported fungal culture positivity of 2.95% (21/713) and mycobacterial positivity of 3.02% (10/331); importantly, only 13 fungal cultures were confirmed as true fungal infections, and mycobacteria grew in four cultures among five infected patients, leading the authors to conclude that routine fungal and mycobacterial cultures may not be necessary in most suspected PJI evaluations [17]. This aligns with Golden et al., who reported that out of 91 mycobacterial cultures ordered for suspected PJIs, only one (1.1%) was positive, which was later deemed a contaminant [12]. Tai et al. further supported this conclusion, finding that out of 3,629 fungal cultures performed, 179 (5%) were positive, and only 34 (1.2%) of 2,923 mycobacterial cultures were positive [18].

Positivity rates may differ in other anatomic sites. In foot and ankle surgeries, Dean et al. observed a 0% positivity rate for AFB cultures and a 4.1% positivity rate for fungal cultures, with only 20% of the positive fungal cultures identified as pathologic [16]. Similarly, in spine irrigation and debridement procedures, Lambrechts et al. found a 4% incidence of fungal infection and a 0.49% rate of AFB infection [9]. Conversely, in revision shoulder arthroplasty, Contreras et al. reported no positive fungal or acid-fast cultures [13]. Kazmers et al. found that 7% of patients undergoing surgical debridement of acute deep infections of the upper extremity had positive atypical cultures [19].

Collectively, these studies underscore that the clinical value of fungal and AFB cultures depends not only on whether organisms grow, but on whether the positive result represents a true and management-relevant infection.

Cost Analysis

Although intraoperative fungal and AFB cultures are infrequently positive in many orthopaedic cohorts, they are commonly obtained, leading to substantial cumulative economic and laboratory resource burden. The economic outcomes reported across the reviewed studies were heterogeneous and included hospital or institutional charges, estimated laboratory or health-system costs, projected cost savings from protocol changes, and resource-utilization measures, such as technologist time. These metrics are not interchangeable and should not be interpreted as direct reimbursement estimates unless explicitly stated by the original study authors. Reported economic and resource-utilization metrics associated with intraoperative fungal and AFB cultures are summarized in Table 2.

**Table 2:** Summary of reported economic outcomes associated with intraoperative fungal and acid-fast bacilli (AFB) cultures in orthopedic surgery Abbreviations: AFB = acid-fast bacilli; PJI = prosthetic joint infection; I&D = irrigation and debridement

Study	Cohort/Context	Economic metric reported	Volume/timeframe	Main findings	Key notes
Lambrechts et al. [9]	Spine irrigation & debridement	Cost-effectiveness estimate/projected cost avoidance comparison	10-year period	Routine fungal cultures: $12,151.58 per patient vs $177,297.64 per missed fungal infection requiring treatment	Suggests routine fungal cultures may be cost-effective in higher-risk spine I&D settings
Golden et al. [12]	Suspected PJI	Estimated laboratory cost, projected cost avoidance, personnel/resource opportunity cost	97 patients; 91 cultures	Estimated costs ~$8,740; potential savings $18,730 over 5 years if routine testing discontinued; ~990 hours of technologist time could be freed	Supports discontinuing routine mycobacterial cultures in most suspected PJI workups
Contreras et al. [13]	Revision shoulder arthroplasty	Institutional charge of fungal/AFB cultures	237 procedures (cultures in 158)	Fungal/AFB cultures accounted for 53.2% of total culture charges	Supports targeted testing given 0% positivity (see Table 1)
Wadey et al. [14]	Revision arthroplasty/PJI evaluation	Projected cost savings of fungal/AFB cultures	253 procedures over 1 year; 446 AFB + 486 fungal cultures	Baseline costs exceeded $200,000/year; protocol projected ~$193,110/year savings (assuming ~300 cases/year) and ~80% reduction in unnecessary AFB/fungal testing	Testing burden included 3-10 hours of labor per episode and $350-$600 per episode; estimated ~$66,067 per positive identified; projected ~$965,550 cost avoidance over 5 years. Protocol: send 1 representative specimen for full workup; save remaining tissue for 7 days and add AFB/fungal only if clinically indicated
Tokarski et al. [15]	Presumed aseptic hip/knee revision arthroplasty	Hospital charges	Single institution, 5-year period	Total charges $1,315,533 (~$263,107/year)	Not a protocol paper; supports a high financial burden in low-yield settings

Wadey et al. evaluated 253 orthopedic procedures over one year, during which 446 AFB and 486 fungal cultures were obtained, resulting in total costs exceeding $200,000 [14]. In presumed aseptic hip and knee revision arthroplasty, Tokarski et al. reported total hospital charges for AFB and fungal cultures of $1,315,533 over a five-year period at a single institution, approximately equating to $263,107 annually [15]. In suspected PJI, Golden et al. reported that routine mycobacterial cultures were not justified given low yield; the estimated costs associated with mycobacterial cultures totaled approximately $8,740 over the study period, with potential savings of $18,730 over five years if routine testing were discontinued [12]. In addition to direct costs, the study highlighted opportunity costs, estimating that eliminating unnecessary mycobacterial cultures could free nearly 990 hours of technologist time, thereby improving laboratory resource allocation without compromising care [12].

While AFB and fungal cultures amount to a significant financial burden on the US healthcare system, they can help prevent incurring even greater financial costs by means of earlier detection and subsequent treatment of these uncommon infections. Lambrechts et al. demonstrated that the cost of obtaining fungal cultures for each patient undergoing spine irrigation and debridement over a 10-year period would be $12,151.58 per patient, compared with an estimated $177,297.64 per missed fungal infection requiring subsequent treatment, suggesting that routine fungal cultures may be cost-effective in this higher-risk setting [9].

Given the lack of standardized protocols across institutions to direct indications and methodology of obtaining intraoperative fungal and AFB cultures in orthopedic surgery, variability in ordering likely contributes to inefficient resource utilization. Instituting a standardized protocol to identify indications and methodology of obtaining fungal and AFB cultures provides a potential source of cost reductions. The protocol developed by Wadey et al. resulted in an 80% reduction in unnecessary AFB and fungal cultures, projecting potential cost savings of approximately $193,110 per year for an institution performing 300 cases annually [14]. Future studies should evaluate protocol-driven approaches across orthopedic subspecialties, incorporating both diagnostic yield and downstream economic outcomes to define when atypical cultures provide sufficient value to warrant routine use.

Limitations

This review has several limitations of note. First, this study was designed as a narrative review rather than a systematic review; accordingly, studies were not selected using a PRISMA-based framework, and no formal risk-of-bias assessment or quantitative meta-analysis was performed. Second, the available literature on intraoperative fungal and AFB culture use in orthopaedic surgery is heterogeneous with respect to study design, patient populations, anatomic sites, indications for culture acquisition, and reported outcome measures, which limits direct comparison across studies. Third, much of the literature included in this review was retrospective and single-institutional, which may reduce generalizability. Fourth, reported outcomes were not standardized across studies, especially with respect to the distinction between raw culture positivity, true infection, contamination, and clinically meaningful infection, which may make interpretation of diagnostic yield more difficult. Finally, the reported financial outcomes and economic data were not standardized, and hospital charges, estimated laboratory costs, projected cost savings, and resource-utilization measures, which are not directly interchangeable, may limit the generalizability of the financial conclusions.

## Conclusions

There are no universally accepted guidelines for obtaining intraoperative AFB cultures in orthopedic surgery, and current practice patterns vary widely. Across the reviewed literature, routine fungal and AFB culture acquisition, particularly in high-volume settings, such as arthroplasty and suspected periprosthetic joint infection, demonstrates low diagnostic yield, with many positive results representing contaminants or findings that do not alter clinical management. Despite this, these cultures contribute substantially to healthcare costs and laboratory resource utilization.

Taken together, the available evidence suggests a selective, indication-driven approach rather than routine testing in all cases. From a practical and microbiologic perspective, fungal and AFB cultures may be most appropriate when atypical organisms are suspected, particularly in immunocompromised hosts, patients with steroid exposure or significant vascular comorbidity, and cases characterized by chronic draining wounds, indolent or recurrent infection, repeated debridement, or culture-negative infection with persistent clinical concern. Implementing standardized, targeted culture protocols may reduce unnecessary testing and associated economic burden while preserving the ability to detect clinically meaningful atypical infections in these higher-risk scenarios.
